# Invasive group A streptococcal infection outbreaks of type *emm*118 in a long-term care facility, and of type *emm*74 in the homeless population, Montréal, Quebec

**DOI:** 10.14745/ccdr.v45i01a03

**Published:** 2019-01-03

**Authors:** PA Pilon, N Savard, J Aho, J Caron, A Urbanek, R Paré, P Le Guerrier, C Savard, K Hammond-Collins, C Dung Tran, R Allard, MC Domingo

**Affiliations:** 1 Secteur Prévention et contrôle des maladies infectieuses, Direction régionale de santé publique, Centre intégré universitaire de santé et de services sociaux du Centre-Sud-de-l’Île-de-Montréal [Infectious Disease Prevention and Control Division, Regional Public Health Directorate, Integrated Health and Social Services University Network for South-Central Montréal], Montréal, QC; 2 École de santé publique [School of Public Health], Université de Montréal, Montréal, QC; 3 Department of Epidemiology, Biostatistics and Occupational Health, McGill University, Montréal, QC; 4 Public Health Agency of Canada, Ottawa, ON; 5 Laboratoire de santé publique du Québec [Quebec Public Health Laboratory], Sainte-Anne-de-Bellevue, QC

**Keywords:** invasive, infection, group A streptococcus, long-term care facility, homeless population

## Abstract

**Background:**

Two invasive group A streptococcus (iGAS) infection outbreaks occurred in Montreal in 2016 and 2017; one in a long-term care facility (type *emm*118) and one in the community, primarily involving homeless people (type *emm*74).

**Objective:**

To describe two recent iGAS outbreaks in Montréal and highlight the challenges in dealing with these outbreaks and the need to tailor the public health response to control them.

**Methodology:**

All cases of iGAS were investigated and the isolates were sent to the laboratory for *emm* typing. In both outbreaks, cases of superficial group A *streptococcus* (GAS) infection were identified, through 1) systematic case detection accompanied by screening for asymptomatic carriers among residents and employees of the long-term care facility and 2) sentinel surveillance among homeless people. Visits were made to community organizations providing homeless services (including shelters) and social networks were analyzed to establish whether there were any links among cases of GAS infection (both invasive and noninvasive) and locations frequented. In both outbreaks, recommendations were made to service providers regarding enhancement of infection prevention and control measures.

**Results:**

In the long-term care facility, five cases of type *emm*118 iGAS were identified over a 22-month period, one of which resulted in death. All residents were screened and no carriers were identified. Among the employees, 81 (65%) were screened and fourcarriers were identified. Of those, one was a carrier of type *emm*118 GAS. All carriers were treated, and subsequent follow-up sampling on three carriers (including the one with *emm*118) was negative.

In the community, 23 cases of type *emm*74 iGAS were detected over a 16-month period, four of which resulted in death. Half of the cases (n=12) were described as homeless, and six others were users of services for the homeless. Sentinel surveillance of superficial infections yielded 64 cultures with GAS, chiefly on the skin, including 51 (80%) of type *emm*74. An analysis of the social networks revealed the large number and variety of resources for the homeless used by the cases. Visits to the community organizations providing homeless services revealed the heterogeneity and precariousness of some of these services, the difficulties encountered in applying adequate health and hygiene measures, and the high degree of mobility amongst those who use these services.

**Conclusion:**

The detection and control of iGAS outbreaks in both long-term care establishments and among community organizations providing homeless services are very complex. An outbreak of iGAS can develop in the background over a long time and be easily overlooked despite cases being admitted to the hospital. *Emm* typing and systematic research of previous cases of iGAS are essential tools for the detection and characterization of outbreaks. Close cooperation among public health agencies, clinical teams, community organizations and laboratories is essential for proper monitoring and the reduction of GAS transmission in the community and health care settings.

## Introduction

The β-hemolytic group A streptococcus (GAS) (*Streptococcus pyogenes*) is a bacterium that is transmitted primarily through droplet inhalation or via skin contact. Sites such as the oropharynx and skin can be asymptomatically colonized. There is a spectrum of infections that can be caused by GAS, ranging from benign infections, such as pharyngitis or impetigo, to invasive infections, such as bacteremia, necrotizing fasciitis or streptococcal toxic shock syndrome (1). The sequencing of a portion of the *emm* gene, which codes for a virulence factor called the M protein, allows the identification of 220 distinct strains, called *emm* types (2).

The risk factors for invasive GAS (iGAS) infections include advanced age, skin lesions or breaks, viral respiratory infections, some chronic diseases such as diabetes, immunosuppression, use of intravenous drugs and excessive use of alcohol (1,3–5).

**Figure 1:**
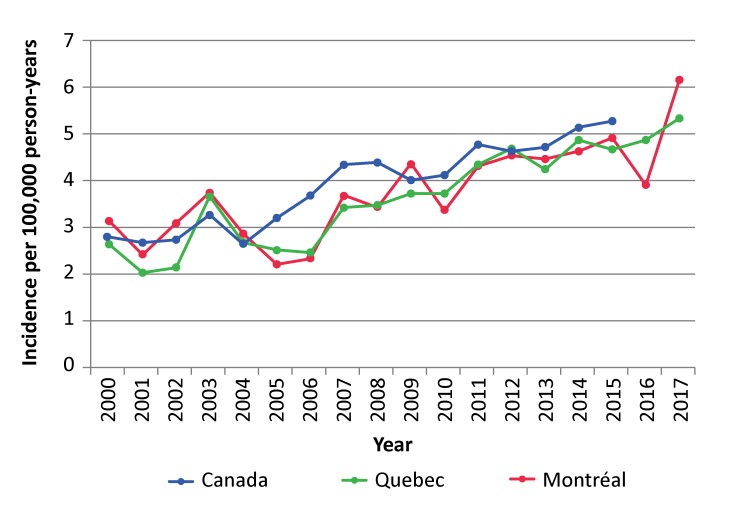
Incidence of invasive group A streptococcal infections for Montréal, the province of Quebec and Canada, 2000–2017 Sources: Public Health Agency of Canada. Number of invasive cases of group A streptococcal infections reported between 1994 and 2015 – Notifiable diseases online (consulted February 21, 2018); Institut national de sant *é* publique du Qu *é* bec. *Rapport hebdomadaire/annuel des maladies à déclaration obligatoire d'origine infectieuse* [Weekly/Annual Report of Infectious Notifiable Diseases] (consulted February 21, 2018)

In Quebec, iGAS is a notifiable disease.The Direction régionale de santé publique (DRSP) [Regional Public Health Department], Centre intégré universitaire de santé et de services sociaux (CIUSSS) du Centre-Sud-de-l’Île-de-Montréal handles such reports for a population base of approximately two million people.Cases of iGAS reported by labsand physicians are systematically investigated by the DRSP to identify the severity of the infection, the associated risk factors and any close contacts who might benefit from prophylaxis as per provincial recommendations (6). Since 2010, isolated strains from sterile sites have been sent to the Laboratoire de santé publique du Québec (LSPQ) [Quebec Public Health Laboratory], then to the National Microbiology Laboratory (NML), which does the *emm* gene typing. An antibiogram is performed to test for sensitivity to penicillin, ceftriaxone, erythromycin, clindamycin and vancomycin. Surveillance statistics show that the incidence of iGAS is on the rise in Montréal, the province of Quebec as a whole and across Canada (**Figure 1**) (6–9).

In 2016 and 2017, the DRSP detected two outbreaks of iGAS in very different populations: in residents of a long-term care facility; and in a homeless population. The goal of this article is to describe these two outbreaks and to illustrate the challenges encountered and the need to tailor responses to the specific affected populations.

## Outbreak in a long-term care facility

### Background

Due to a combination of host- and environment-related factors, elderly people in a long-term care environment are at higher risk of contracting and dying from iGAS (10,11). Several iGAS outbreaks, occurring mostly over periods of a few months, have been reported in this type of setting within Canada and elsewhere in the world (3,12–15).

In Quebec, the recommendation is to conduct a 30-day surveillance after any iGAS case is reported in a long-term care resident. If the iGAS case is severe (meningitis, pneumonia, soft-tissue necrosis, toxic shock or death), antibiotic prophylaxis is given to all close contacts and a retrospective investigation is conducted for the previous 30 days (6). If a high number of cases are identified, the recommendation is to look for asymptomatic carriers and carry out a prospective surveillance of invasive and noninvasive GAS infections (6,7).

### Methodology

#### Detection of the outbreak

 

In July and August 2016, two cases of type *emm*118 iGAS were reported to the DRSP by a long-term care facility that accommodated approximately 200 residents. The public health investigator noted that other cases of iGAS had occurred in the same long-term care facility over the two previous years. The period covered by the investigation was therefore expanded beyond the routinely recommended 30-day retrospective period.

#### Definition of a case

 

A confirmed case of iGAS is defined as being a long-term care facility employee or resident in whom type *emm*118 GAS was isolated from a normally sterile site any time after January 1, 2014. Asymptomatic carriers or superficial GAS infections are defined as being an employee or resident in whom type *emm*118 GAS was isolated from a non-sterile site (oropharynx, wound) any time after January 1, 2014.

#### Search for cases

 

All cases of iGAS reported to the DRSP by this long-term care facility were reviewed. Lab results positive for GAS (as of January 1, 2014) for residents of the long-term care facility and patients of its referring hospital were retrospectively reviewed to identify cases of iGAS that may not have been reported as well as cases of noninvasive GAS infection. Active surveillance began in August 2016 and continued until April 2017 to detect type *emm*118 GAS, whether invasive or not.

Screening for asymptomatic carriers was proposed to all residents and health care staff via throat swabs and, if applicable, wound swabs. This screening offer started in the summer of 2016 and continued for 12 weeks.

#### Microbiological analyses

 

The NML performed *emm* typing of isolates from both sterile and non-sterile sites. While awaiting the results of *emm* typing, the LSPQ conducted pulsed-field gel electrophoresis (PFGE) on isolates taken from iGAS cases to confirm similarities between invasive strains.

#### Data analysis

 

The descriptive analyses and the epidemic curve were derived using Microsoft™ Excel 2010 software.

### Results

Five confirmed cases of type *emm*118 iGAS were identified via the systematic review of cases reported to the DRSP. Pulsed-field gel electrophoresis analyses indicated the same profile for these strains. All type *emm*118 isolates were sensitive to all antibiotics tested. A review of patient records in the facility and prospective surveillance revealed no additional cases.

**Figure 2:**
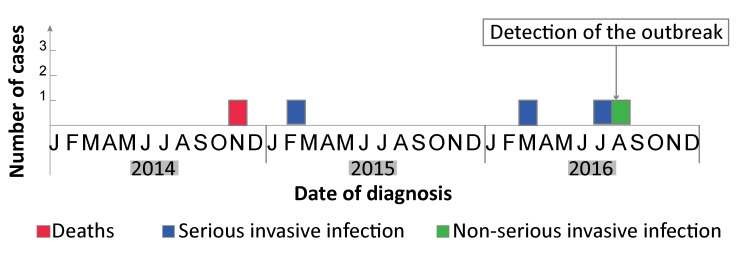
Epidemic curve of cases of invasive type *emm*118 group A streptococcal infection in a Montréal long-term care facility, 2014–2016 (n=5)

All cases of type *emm*118 iGAS were found in men 71 to 84 years of age. All had comorbidities, notably kidney failure, chronic obstructive pulmonary disease, immunosuppression or cirrhosis. None had any chronic wounds, skin infections or skin diseases. Four cases presented a serious infection (one death from streptococcal toxic shock and three cases of pneumonia) and one case presented with bacteremia. All of the cases occurred between November 2014 and August 2016 (**Figure 2**). No close contacts between cases of type *emm*118 iGAS were identified.

All residents were screened. None of them were GAS carrier. Among employees, 81/125 (65%) were tested: four were GAS carriers, one of whom was *emm*118-positive. This particular person was working in the facility when the five cases of iGAS were reported. All employees who were carriers were withdrawn from work for the first 48 hours of treatment (10 days of oral penicillin V, amoxicillin or cefadroxil). The success of the treatment was ascertained by an oropharyngeal culture from three of the four employees, including the carrier of the *emm*118 strain (6). No further cases were identified in the subsequent six months.

## Outbreak in a homeless population

### Background

Due to a combination of risk factors, homeless individuals are at greater risk of iGAS (16,17). They are over-represented among reported cases in Montréal (*unpublished data*), although no outbreak had been previously documented among this population in Montréal. Elsewhere, outbreaks of iGAS have been reported among intravenous drug users (18–23) and, more recently, among homeless populations (24–28).

### Methodology

#### Detection of the outbreak

 

Between March and May 2017, the DRSP received reports of two cases of iGAS at the same shelter. These two instances led to a review of all cases reported among homeless people in 2017. A greater number of cases (n=7) had occurred in this population since January 2017, compared with the same period in the preceding year (n=3). The first five cases for which the *emm* type was identified were of *emm*74 type. This *emm* type had not previously been reported in Montréal.

#### Definition of cases

 

An iGAS outbreak case was defined as a person living in Montréal, with isolation of type *emm*74 GAS from a normally sterile site between March 1, 2017 and July 31, 2018. An outbreak case with superficial (noninvasive) infection was defined as being a person identified by the sentinel surveillance initiated during the outbreak and having at least one culture from a non-sterile site testing positive for type *emm*74 GAS.

#### Investigation of cases

 

Following detection of the outbreak, the iGAS cases were again investigated to identify which homeless services and shelters were used during the month preceding the appearance of symptoms or, if accurate timing was impossible, the generally frequented places. For new cases, several investigations were conducted in person with the cases, in order to obtain more comprehensive information about their risk factors, places frequented and close contacts.

#### Surveillance of noninvasive infections

 

A sentinel surveillance of cases of superficial infection was initiated in July 2017. The main clinical teams working with the homeless population were asked to look for any wounds that seemed infected, to swab these wounds for culture and to fill out a short survey documenting the homeless services and shelters used in the two preceding weeks. The cultured GAS strains were sent to the LSPQ, then to the NML for *emm* typing.

#### Data analysis

 

Descriptive statistics and the comparison with sporadic cases of iGAS in Montréal were performed with IBM Cognos Business Intelligence™ 10.2.2 software (International Business Machines Corp., Armonk, New York, US), Microsoft Excel 2010 and Stata™ 15 (StataCorp, College Station, Texas, US). Social networks were analyzed using the Pajek 5.04 application. The resulting network includes cases of iGAS, their close contacts as defined in Quebec guidelines (6), the cases of superficial GAS infection detected via sentinel surveillance and the places frequented (i.e., shelters and other residential locations, day centres, clinical services for the homeless and gathering places).

### Intervention

The DRSP worked in close cooperation with the *Service régional en itinérance* [Regional Homeless Service], clinical teams working with the homeless, and several dozen community agencies that operate shelters, day centres and other services for the homeless.

In June, an alert was sent out to the health care network and recommendations for basic hygiene measures were forwarded to community organizations providing services to the homeless. Recommendations focused on the early detection of infected wounds, swabbing them for culture and on their treatment. In July the sentinel surveillance system for monitoring wounds was established.

Throughout June and July, the DRSP visited nine shelters and three day centres to assess the situation and issue more specific recommendations to decrease the transmission of infections, to facilitate hygiene and cleanliness, and to control infestations by ectoparasites, as these could lead to wounds initiated from scratching. In October, follow-up visits were conducted to evaluate the implementation of recommendations and to distribute a reader-friendly information poster to inform people using these homeless services of the ways they can protect themselves. As recommended by provincial guidelines (6), antibiotic prophylaxis for people who had close contact with severe cases of iGAS was offered whenever possible.

### Results

The first iGAS case of this outbreak occurred in March 2017. The outbreak was declared over on July 31, 2018, after eight consecutive months with no excessive cases of iGAS among homeless services users. The outbreak had 23 cases of iGAS, 19 of which were reported between March and November 2017. The cases of iGAS included 14 men and nine women from 34 to 80 years of age (median=54).

The most common clinical presentations were soft-tissue infections (n=14, including five cases of necrotizing fasciitis) and bacteremia (n=7). All were hospitalized, eight went into toxic shock and four died. Half of the cases (n=12) were homeless when the infection occurred, six were not homeless but had used homeless services, and three had an epidemiological link to this population. Two subjects had no links to this population. Twelve people consumed excess alcohol and three used intravenous drugs.

The outbreak’s iGAS cases were significantly different from the sporadic cases reported in Montréal during the same period (**Table 1**). All of the *emm*74 iGAS isolates were sensitive to all of the antibiotics tested.

**Table 1:** Comparison of cases from the invasive type *emm*74 group A streptococcal infections with other cases from the same period

Characteristics	Type *emm*74 March 2017 to July 2018		Other *emm* type or unknown March 2017 to July 2018		*P* value^a^
	N=23	%	N=167	%	
Median age (years)	54	N/A	46	N/A	N/A
Male	14	60.9	93	55.7	0.662
Hospitalized	23	100.0	153	91.6	0.225
Death	4	17.4	10	6.0	0.072
Infection types					
Soft tissue^b^	14	60.9	64	38.3	0.045^d^
Respiratory^c^	3	13.0	28	16.8	1.000
Bacteremia	7	30.4	35	21.0	0.295
Toxic shock	8	34.8	19	11.4	0.007^d^
Risk factors					
Homelessness	12	52.2	8	4.8	<0.001^d^
Drugs	4^e^	17.4	11	6.6	0.090
Alcoholism	12	52.2	17	10.2	<0.001^d^
Diabetes	5	21.7	20	12.0	0.195
Chronic pulmonary diseases	6	26.1	11	6.6	0.008^d^

Abbreviations: N/A, not applicable; N, total number; <, inferior to

^a^ Fisher’s exact test for comparison of cases of invasive group A streptococcal infections type *emm*74 and those of other or unknown *emm* type

^b^ Soft tissue infections include soft tissue necrosis, fasciitis, myositis and/or cellulitis or erysipelas

^c^ Respiratory infections include pneumonia or other pulmonary manifestation and/or pharyngitis/tonsillitis

^d^ Statistically significant (α = 0.05)

^e^ Including intravenous and oral drugs

**Figure 3:**
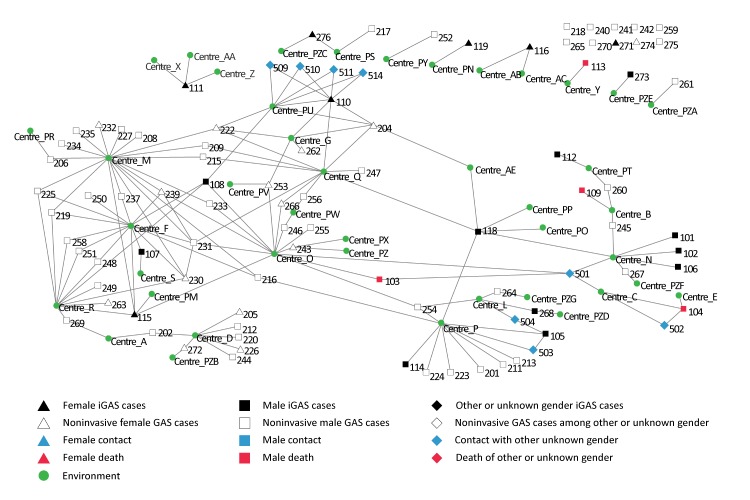
Analysis of social networks for cases of invasive and noninvasive *emm*74^a^ type group A streptococcal infections Abbreviations: GAS, group A streptococcus; iGAS, invasive group A streptococcus ^a^ The network illustrated excludes two cases of iGAS from the outbreak that were epidemiologically unrelated to the homeless population. It includes one case of type *emm*74 iGAS in a non-Montrealer who was epidemiologically related to the homeless population

Of a total of 156 specimens submitted, sentinel surveillance detected 63 wounds and one throat that had been infected or colonized by GAS, of which 51 (80%) were type *emm*74. An analysis of the social networks revealed eight components, the chief of which linked 68% of iGAS cases and 81% of superficial infection cases (**Figure 3**). Some locations had been frequented by a greater number of cases (four cases of iGAS for one location, 17 noninvasive cases for another), but no single environment was directly linked with a majority of cases.

For the nine severe cases of iGAS who used homeless services or were epidemiologically related, only four close contacts could be identified for antibiotic prophylaxis. The community organizations had been alerted to the importance of prevention measures, but the necessary resources were not always available, which introduced some variability with respect to the application of prevention and treatment measures. The main challenges encountered were the frequency of housekeeping, the cleaning of beds and bed linens, the laundering of clothing and the availability of a change of clothing. Noteworthy improvements were observed in some organizations during follow-up visits.

## Discussion

Managing these two iGAS outbreaks in two very different contexts identified both common and context-specific challenges. Detecting the outbreak proved to be a challenge in both contexts. For the long-term care outbreak, the length of time between cases led to a 21-month delay between the appearance of the first case and the detection of the outbreak. For the outbreak among the homeless population, detection was more timely but could have been delayed had two related cases not been reported in the same shelter. The absence of specific surveillance by *emm* type was a limiting factor in the detection of both outbreaks.

The long-term care outbreak was characterized by its long-lasting nature and the presence of the same *emm* type in an employee. In the absence of close contact among the different cases of iGAS, the most likely assumption is transmission via an asymptomatic carrier, possibly the *emm*118 GAS-positive employee, although this person could also have been a secondary case, with the primary case being an unidentified carrier. The work of long-term care personnel in identifying and dealing with similar future outbreaks could be facilitated with the development of an operational protocol for screening, treatment and follow-up of screened employees. The DRSP’s recommendation to long-term care facility was to continue prospective surveillance of GAS infections for a minimum of six months after the last identified case, even though the optimal timing for prospective surveillance is currently unknown (29). Of note, no further cases of iGAS infection were detected at the long-term care facility during this additional surveillance period. Enhancement of infection prevention and control measures, and detection and treatment of the *emm*118 GAS carrier, may have contributed to this outcome, as it has been reported in other outbreaks (5,12,29,30).

For the homeless population outbreak, analysis of the social network and sentinel surveillance revealed that the outbreak was not limited to one specific setting. Rather, the strain involved was found to be circulating throughout the homeless population. This population is inherently mobile: homeless people can sleep in different places and call upon different resources, which would account for widespread transmission. This mobility creates a daunting challenge in terms of intervention. Other challenges encountered included limitedhuman and financial resources available for the application of recommendations regarding hygiene and cleanliness, mental health issues among the users, which make it difficult to apply personal hygiene measures and treat infestations, the difficulty of accessing proper care and the wide variety of community organizations involved. In addition, there is no way of assessing the effectiveness of the public health interventions and distinguishing their effect from the natural evolution of the outbreak.

These iGAS outbreaks have identified the need for improved detection methods. *Emm* typing of iGAS and systematic search for previous cases of iGAS are essential tools for detecting and characterizing outbreaks. These outbreaks have also prompted the development of more effective ways of collaboration between clinical partners, community organizations and laboratories to pave the way for setting-specific interventions. The development of intervention strategies to identify and control iGAS outbreaks in vulnerable populations, whether in the community or in a health care setting, is especially relevant in a context where the incidence of iGAS is on the rise in Canada.
